# Efficacy of a Self-Guided Transdiagnostic Intervention for Adults With Anxiety and Depression: Randomized Controlled Trial

**DOI:** 10.2196/79759

**Published:** 2025-10-23

**Authors:** Walter Staiano, Christine E Callahan, Michelle Davis, Leah Tanner, Sarah Kunkle, Jenna Glover, Jon Kole, Neeru Bakshi, Marco Romagnoli, Ulrich Kirk

**Affiliations:** 1 Department of Physical Education and Sport University of Valencia Valencia Spain; 2 Department of Psychology University of Southern Denmark Odense Denmark; 3 Headspace Inc Santa Monica, CA United States; 4 Department of Psychiatry and Human Behavior Brown University Providence, RI United States; 5 Fralin Biomedical Research Institute Roanoke, VA United States

**Keywords:** cognitive behavioral therapy, anxiety, depression, mindfulness, meditation, mobile health, smartphone intervention, Headspace, Generalized Anxiety Disorder-7, GAD-7, Patient Health Questionnaire-8, PHQ-8

## Abstract

**Background:**

Anxiety and depressive disorders affect hundreds of millions globally, with substantial barriers limiting access to evidence-based treatments like cognitive behavioral therapy. Digital mental health interventions offer potential solutions to improve access to support. However, evidence of efficacy through randomized controlled trials is essential for clinical adoption.

**Objective:**

This study evaluated the efficacy of the Headspace Anxiety and Depression Program, a digitally delivered 21-session intervention grounded in the Unified Protocol that integrates cognitive behavioral and mindfulness-based strategies to target common drivers of emotional disorders, compared to a waitlist control group. Primary aims were to assess reductions in anxiety (Generalized Anxiety Disorder-7 [GAD-7]) and depression (Patient Health Questionnaire-8 [PHQ-8]) symptoms. Secondary aims examined improvements in sleep quality (Pittsburgh Sleep Quality Index), perceived stress (Perceived Stress Scale-10), mindfulness (Mindful Attention Awareness Scale), and overall well-being (Warwick-Edinburgh Mental Well-Being Scale), as well as clinical remission and treatment response rates.

**Methods:**

A fully remote, 2-arm parallel-group randomized controlled trial was conducted with 168 participants (aged ≥18 years) who had clinically significant anxiety (GAD-7≥10) or depression symptoms (PHQ-8≥10). Participants were randomized to either the Headspace Anxiety and Depression Program (n=84) or waitlist control (n=84). The intervention consisted of 21 daily sessions (5-10 minutes each) delivered via the Headspace app. Primary (GAD-7 and PHQ-8) and secondary outcomes (Pittsburgh Sleep Quality Index, Perceived Stress Scale-10, Mindful Attention Awareness Scale, and Warwick-Edinburgh Mental Well-Being Scale) were assessed at baseline, postintervention, and a 3-week follow-up using intention-to-treat analysis with mixed-model ANOVAs.

**Results:**

Study retention was high, with the majority (154/168, 91.7%) of participants completing the 3-week follow-up visit, and no serious adverse events were reported. Intervention adherence was high, with 82.1% (69/84) in the intervention group completing all 21 sessions. Significant group×time interactions were observed for both primary outcomes (*P*<.001). The Headspace group showed substantial reductions in anxiety symptoms (GAD-7: 34.5% reduction from baseline: mean 11.9, SD 2.8 to follow-up: mean 7.8, SD 2.3; η^2^_p_=0.350) and depression symptoms (PHQ-8: 33.9% reduction from baseline: mean 12.1, SD 2.8 to follow-up: mean 8.0, SD 2.0; η^2^_p_=0.370), while the control group did not show significant reductions. Combined anxiety and depression remission rates were significantly higher in the Headspace group (18/84, 21.4%) versus controls (7/84, 8.3%; *P*=.03), as were treatment response rates (23/84, 27.3% vs 2/84, 2.3%; *P*=.03). Participants in the Headspace group also demonstrated significant improvements in secondary outcomes, including sleep quality (30.2% improvement), perceived stress (13.2% reduction), mindfulness (10.3% increase), and mental well-being (10.7% increase).

**Conclusions:**

The Headspace Anxiety and Depression Program demonstrated significant efficacy in reducing anxiety and depression symptoms comparable to established treatments. Benefits were sustained at the 3-week follow-up. These findings support the potential of evidence-based transdiagnostic digital mental health interventions to address critical gaps in mental health care delivery.

**Trial Registration:**

ClinicalTrials.gov NCT06434883; https://clinicaltrials.gov/study/NCT06434883

## Introduction

Anxiety and depressive disorders represent 2 of the most prevalent and debilitating mental health conditions globally, affecting hundreds of millions of individuals worldwide and imposing substantial personal, social, and economic burdens [1]. In the United States, approximately 31% of adults report experiencing an anxiety disorder at some point in their lives, with 20% reporting anxiety symptoms within the past year [2]. Additionally, 13% of adolescents and adults experience depression, with 88% reporting functional impairment in work, home, and social activities [3]. For the purposes of this study, we focus on generalized anxiety symptoms as measured by the Generalized Anxiety Disorder-7 (GAD-7), which assesses worry, nervousness, irritability, and difficulty relaxing—core features of generalized anxiety disorder that often co-occur with depression and significantly impair daily functioning. The comorbidity between these conditions is particularly concerning, with approximately 50%-60% of those with anxiety symptoms also experiencing depression symptoms [4], which may further worsen mental health and overall quality of life.

Cognitive behavioral therapy (CBT) has been extensively validated as an effective treatment for both anxiety and depression disorders [5], with both in-person and teletherapy-delivered CBT showing consistent outcomes [6]. However, both in-person and teletherapy CBT face substantial barriers including insurance coverage limitations, treatment costs, uncertainty about where to seek treatment, lack of access to qualified therapists, and scheduling inconvenience due to workday constraints [7]. While teletherapy has made CBT more accessible, these barriers continue to limit the accessibility of evidence-based treatments for addressing the growing mental health needs of the population. Recognizing these barriers, empirically validated components of psychological interventions that were once delivered solely via CBT have increasingly been translated into digital mental health interventions (DMHIs) [8,9]. These DMHIs are often delivered via app or online, completed asynchronously, and require less time and monetary commitment than CBT. The emergence of the DMHIs represents a paradigm shift in treatment accessibility and delivery, offering potential solutions to overcome traditional barriers to care. Smartphone apps are one form of digital treatment delivery receiving substantial attention, with mental health apps for depression and anxiety being among the most widely downloaded categories of health apps [10]. The ubiquity of smartphone technology presents unprecedented opportunities to deliver evidence-based interventions directly to individuals in need, transcending traditional barriers of geography, cost, scheduling constraints, and stigma associated with help-seeking [11].

Recent meta-analytic evidence has provided increasingly robust support for the efficacy of DMHIs [12], where they showed that apps have overall small but significant effects on symptoms of depression (n=33,567; *g*=0.28) and generalized anxiety (n=22,394; g=0.26) compared to control conditions [13-15]. While evidence exists on the impact of DMHIs, there is still a critical shortage in the understanding of smartphone-delivered mental health interventions, as only 3% of apps that claimed to offer a therapeutic treatment for anxiety and depression had published peer-reviewed research to back up their assertions [16]. Additionally, engagement and adherence represent persistent challenges in DMHIs. Studies have demonstrated that engagement with DMHIs varies widely, with naturalistic use often showing considerable attrition even among those who initially download apps [17]. A lack of engagement with mental health apps has been reported as a key limitation for realizing the potential of apps to broadly disseminate mental health treatments.

This study addresses several critical gaps in the existing literature by conducting a randomized controlled trial (RCT) examining the efficacy of the Headspace Anxiety and Depression Program. The DMHI is grounded in the Unified Protocol (UP) [18], a transdiagnostic treatment that integrates cognitive behavioral and mindfulness-based strategies to target common drivers of emotional disorders, such as avoidance and inflexible thinking, seen across conditions like anxiety and depression. The Headspace Anxiety and Depression Program is a self-guided program consisting of 21 daily 5- to 10-minute sessions. This study evaluated the efficacy of the Headspace Anxiety and Depression Program by addressing both primary symptoms of depression and anxiety as well as secondary outcomes including sleep quality, perceived stress, mindfulness, and overall well-being. Furthermore, the study explored treatment response and remission rates, in addition to symptom reduction measures, to allow for the evaluation of clinical outcomes. The Headspace Anxiety and Depression Program is grounded in a transdiagnostic framework based on the UP, which recognizes that anxiety and depression share common underlying mechanisms, particularly emotion dysregulation and maladaptive coping strategies. The program operates through several interconnected mechanisms within this UP framework. First, mindfulness-based components enhance emotional awareness and reduce automatic reactivity to distressing thoughts and feelings. Second, cognitive flexibility exercises help participants recognize and modify maladaptive thought patterns common in both anxiety and depression. Third, behavioral experiments and exposure components directly address avoidance behaviors that maintain both conditions. The integration of these approaches within the UP transdiagnostic framework targets shared vulnerability factors such as neuroticism and emotion dysregulation that underlie both anxiety and depressive disorders. While anxiety and depression have distinct symptomatic presentations, the transdiagnostic approach assumes that they share core underlying mechanisms, particularly related to emotion regulation difficulties, cognitive biases, and behavioral avoidance. The intervention addresses these common mechanisms while allowing for individual adaptation to specific symptom presentations. We hypothesized that participants using the Headspace Anxiety and Depression Program would demonstrate significantly greater reductions in depression and anxiety symptoms compared to a waitlist control group, with effect sizes comparable to those reported in recent meta-analyses (Cohen *d*≥0.26-0.28). Secondary hypotheses predicted that the intervention would also yield improvements in sleep quality, perceived stress levels, mindfulness, and overall well-being.

## Methods

### Study Design

This study was an app-based, 2-arm parallel-group RCT comparing the Headspace Anxiety and Depression Program (intervention) to a waitlist control group. The study followed CONSORT (Consolidated Standards of Reporting Trials) 2010 guidelines for reporting parallel group randomized trials [19]. A completed CONSORT checklist is provided in Multimedia Appendix 1.

### Ethical Considerations

Study procedures were approved by the institutional review board of Virginia Tech (#21-295), and the study was preregistered (ClinicalTrials.gov NCT06434883). Participants were asked to sign an electronic consent form prior to enrolling in the study and were informed that they could opt out of the study at any time. All data were deidentified prior to analysis. Participants were compensated for taking part in the study using the following compensation structure: US $50 for completing the baseline assessment, US $50 for completing the postintervention assessment, and US $50 for completing the follow-up measures.

### Participants

Overall, 514 potential participants were assessed for eligibility, with 168 meeting the inclusion criteria and enrolling in the study. Inclusion criteria were assessed through a structured online eligibility screening process. All potential participants completed the Patient Health Questionnaire-8 (PHQ-8) and GAD-7 as part of this screening to determine eligibility. Inclusion required (1) clinically significant symptoms of depression or anxiety as determined by validated cutoff scores (PHQ-8≥10 or GAD-7≥10) [20,21], (2) no prescription medication for anxiety or depressive symptoms or a stable dose for ≥4 weeks, (3) 18 years or older, (4) located in the United States, and (5) access to a compatible smartphone (Android or iOS). Exclusion criteria, also assessed by self-report during the eligibility screening phase, were (1) severe depression (PHQ-8>20) or risk of self-harm (positive response to the self-harm screening question, “The thought of harming myself has occurred to me.”); (2) not maintaining a stable dose of psychotropic medication for ≥4 weeks prior to enrollment; (3) psychiatric hospitalizations within the past 6 months; (4) self-reported diagnosis of any of the following conditions: schizophrenia, psychosis, bipolar disorder, seizure disorder, substance use disorder, recent trauma to the head or brain damage, severe cognitive impairment, serious physical health concerns necessitating surgery, or pregnancy; and (5) having completed CBT or other psychotherapy that included self-monitoring and cognitive or behavioral exercises delivered by a licensed therapist in the past 6 months. Individuals reporting self-harm risk were referred to mental health resources (National Alliance on Mental Illness helpline) per safety protocol.

### Study Procedures

Participants were recruited from across the United States via targeted social media advertisements on Facebook and Instagram. The advertisements featured brief descriptions of the study purpose (evaluating a digital mental health program for anxiety and depression), eligibility criteria, and compensation details. Potential participants accessed a secure web-based platform using REDCap (Research Electronic Data Capture; Vanderbilt University) hosted at Virginia Tech. The platform facilitated all study procedures including eligibility screening, electronic informed consent, randomization, and data collection. Apart from app use data, all data were collected and managed through the REDCap platform. This infrastructure enabled fully remote participation via a web-enabled device. App use data was automatically captured and stored securely within Headspace’s servers.

Following the initial overview of the study, potential participants completed an eligibility screening to assess inclusion and exclusion criteria. After eligibility screening, the research team assessed the input from potential participants and notified eligible participants with instructions on how to proceed. Subsequently, eligible participants reviewed the consent document through the research platform, with an option to download it as a PDF before providing electronic consent.

Enrolled participants completed all baseline outcome assessments. Specifically, participants completed the primary outcome measures (PHQ-8 and GAD-7) and secondary outcome measures Perceived Stress Scale-10 (PSS-10) for measuring self-reported stress [22], Pittsburgh Sleep Quality Index (PSQI) for measuring sleep quality [23], Mindful Attention Awareness Scale (MAAS) for measuring mindfulness [24], and Warwick-Edinburgh Mental Well-Being Scale (WEMWBS) for measuring mental well-being [25].

Following baseline data collection, participants were randomized to either the intervention (Headspace Anxiety and Depression Program) or the waitlist control group. The randomization sequence was created by the MATLAB randi() function to provide an array of “1” or “2.” The randomization sequence was uploaded to the REDCap study platform to allow for allocation concealment and automated randomization of enrolled participants. The sequence was concealed until analysis; however, 1 member of the research team was unblinded to track adherence and provide technical assistance. The research team emailed participants an auto-generated email based on their group allocation. Participants randomized to the intervention group received instructions on how to download and access the Headspace app including instructions about the Anxiety and Depression program. Participants were provided with written instructions to only access the Anxiety and Depression Program in the Headspace app during the study. Participants were provided with details about how to contact the research team if they experienced technical challenges related to app setup and app use. None of the participants in the study reported technical challenges when setting up the app or reported experiencing technical challenges during the study.

Participants responded to outcome measures at the end of the intervention period (postintervention assessment). Participants in the intervention group were asked not to access the Headspace Anxiety and Depression Program from the time of postintervention assessment until completion of the 3-week follow-up assessment to allow for evaluation of intervention durability without ongoing exposure. This protocol decision was made to maintain scientific rigor in assessing sustained effects, though we acknowledge this created an ethical tension between research methodology and participant benefit. This temporary limitation was included in the study protocol, approved by the institutional review board, and participants were informed of this during the consent process. Participants were advised that they could access other mental health resources if needed during this period. Follow-up outcome measures were collected 3 weeks following the postintervention assessment.

### Safety

Safety was assessed through monitoring of adverse events throughout the study period. Participant responses indicating clinical deterioration or elevated risk were automatically flagged in the REDCap platform for review by the research team.

### Headspace Anxiety and Depression Program (Intervention)

The Headspace Anxiety and Depression Program integrates CBT and mindfulness techniques similar to the approach used in the UP, a transdiagnostic approach to reducing anxiety and depression symptoms (Multimedia Appendix 2). The 21-session program (5-10 minutes per session, intended for daily completion) includes education and skill-building in 4 core areas: mindful emotional awareness (cultivating nonjudgmental recognition of thoughts, emotions, and behaviors that contribute to anxiety and depression), behavioral experiments (testing new actions in response to strong emotions), cognitive flexibility (shifting unhelpful thinking patterns), and emotional exposure (reducing avoidance of distressing emotions and situations; Multimedia Appendix 3). The theoretical framework underlying this intervention is based on the principle that anxiety and depression share common underlying mechanisms, particularly emotion dysregulation, cognitive inflexibility, and behavioral avoidance. The program systematically addresses these through a structured progression: early sessions build mindful awareness of emotional patterns, middle sessions introduce cognitive and behavioral tools for managing difficult emotions, and final sessions consolidate these skills for long-term application (Multimedia Appendix 4). Sessions 1-5 are focused on building awareness through mindfulness skills, values exploration, and self-monitoring. Sessions 6-14 introduce cognitive, behavioral, and emotional regulation strategies, emphasizing active experimentation and skill development; and sessions 15-21 reinforce these techniques through continued practice, with a focus on problem-solving, behavior change, and relapse prevention. Sessions 1-14 comprise the program’s core content and are considered the minimum effective dose, as all key techniques are introduced during this phase. In contrast to other CBT programs, which often run 30-60 minutes per session [26], the content is delivered through brief daily videos (5-10 minutes) in the Headspace app, each paired with a simple, actionable suggestion (or “daily action”) to help users apply the day’s learning (eg, trying a new wind-down routine). To increase relatability and engagement, the program features animations and real-world video segments with mindfulness teachers, clinical experts, and individuals sharing personal experiences with anxiety and depression. This approach was intended to help users see their experiences reflected in the content and better understand how techniques can be adapted to apply them to everyday life. Participants received automated in-app reminders, but no additional human support beyond the technical assistance contact information provided at enrollment.

### Waitlist Control

Participants in the waitlist control group were instructed to maintain their regular daily routines and activities throughout the study period. They received no intervention content during this time but completed all assessment measures at identical time points to the intervention group. Control participants were provided with contact information for mental health resources and crisis support services. To minimize potential differential attrition, waitlist participants were informed during consent that they would receive full access to the Headspace Anxiety and Depression Program immediately following the study completion.

### Anxiety and Depression Symptoms (Primary Outcomes)

All questionnaires were collected at baseline, postintervention, and 3-week follow-up. Depression and anxiety symptom severity were assessed as primary outcomes using the PHQ-8 and the GAD-7, respectively. The PHQ-8 is an 8-item measure assessing depression symptoms (total score range 0-24; higher scores indicate greater symptom severity; Cronbach α=0.83) [19]. A score ≥10 indicates moderate depression. The GAD-7 is a 7-item measure assessing anxiety symptoms (total score range 0-21; higher scores indicate greater symptom severity; Cronbach α=0.89) [21]. A score ≥10 indicates moderate anxiety. Treatment response was defined as a ≥50% reduction in symptoms from baseline to the 3-week follow-up time point on PHQ-8 or GAD-7. Remission was defined as an absolute score <5 at the 3-week follow-up time point on PHQ-8 or GAD-7.

### Secondary Outcome Measures

Perceived stress was assessed using the PSS-10 (total score range 0-40; higher scores indicate higher perceived stress; Cronbach α=0.85) [22]. Sleep quality was assessed using the PSQI (total score range 0-21; higher scores indicate higher levels of sleep difficulties; Cronbach α=0.83) [23]. Mindfulness was assessed using the MAAS (total score range 15-90; higher scores indicate higher levels of trait mindfulness; Cronbach α=0.89) [24]. Mental well-being was assessed using the WEMWBS (total score range 14-70; higher scores indicate greater mental well-being; Cronbach α=0.91) [25]. Adherence to the Headspace Anxiety and Depression Program was obtained from in-app user data for each participant. Adherence or app use was calculated by summing the total number of completed sessions. Current psychotropic medication use was assessed at baseline and follow-up and included as a potential moderator in exploratory analyses.

### Power Analysis

The sample size calculation was conducted using G*Power [27]. The study aimed to recruit 168 participants, with 84 randomized to each arm and with a 20% attrition rate. The sample size was determined based on an estimated medium effect (0.4-0.5) on postintervention scores between the intervention and waitlist arms, with 90% power, a 1:1 allocation ratio, and with α set at .05. The effect size is based on recent studies of smartphone-delivered psychological treatment for anxiety versus waitlist, which demonstrated a small to moderate between-group postintervention effect on anxiety symptoms when compared to any active or waitlist or inactive control condition [19,28].

### Statistical Analysis

All analyses were conducted using the intention-to-treat approach. For the intention-to-treat analysis, we used multiple imputations to handle missing outcomes (11/168, 6.6%; values were missing at postintervention and follow-up). Multiple imputations (10 imputations) were used to achieve a relative efficiency of 99%, ensuring in-range values.

Assumptions of statistical tests for normal distribution and sphericity of data were checked using the Shapiro-Wilk test, histograms, quantile-quantile plots, and box plots. A series of mixed 2 (groups: intervention and waitlist control)×3 (time: preintervention, postintervention, and follow-up) ANOVAs was performed on GAD-7, PHQ-8, PSQI, PSS-10, MAAS, and WEMWBS. Covariates for elevated GAD-7 and PHQ-8 were included for potential confiding effects. To examine clinical end points, logistic regression analyses were conducted to test differences in treatment response and remission using the cutoff scores described earlier on GAD-7 and PHQ-8 across baseline and follow-up. Logistic regression analyses on remission and response status were performed to compare the 2 groups (intervention and control) at 3-week follow-up (outcome variables) in relation to preintervention values (covariates), without stepwise regression.

All significant interactions were followed up with relevant corrected pairwise comparisons using the Bonferroni method for simple main effects within each group. Where no significant interactions were found, the main effects of time and group were reported. Significance was set at .05 (2-tailed) for all analyses, and ANOVA effect sizes were calculated as partial eta squared (η^2^_p_), with 0.02, 0.13, and 0.26 indicating small, medium, and large effects, respectively. Data analysis was conducted using SPSS (version 27; IBM Corp). Statistical analyses were performed by a member of the research team who was not involved in the intervention delivery. The analyst was blinded to group allocation until completion of primary outcome analyses.

## Results

### Overview

**Figure 1:**
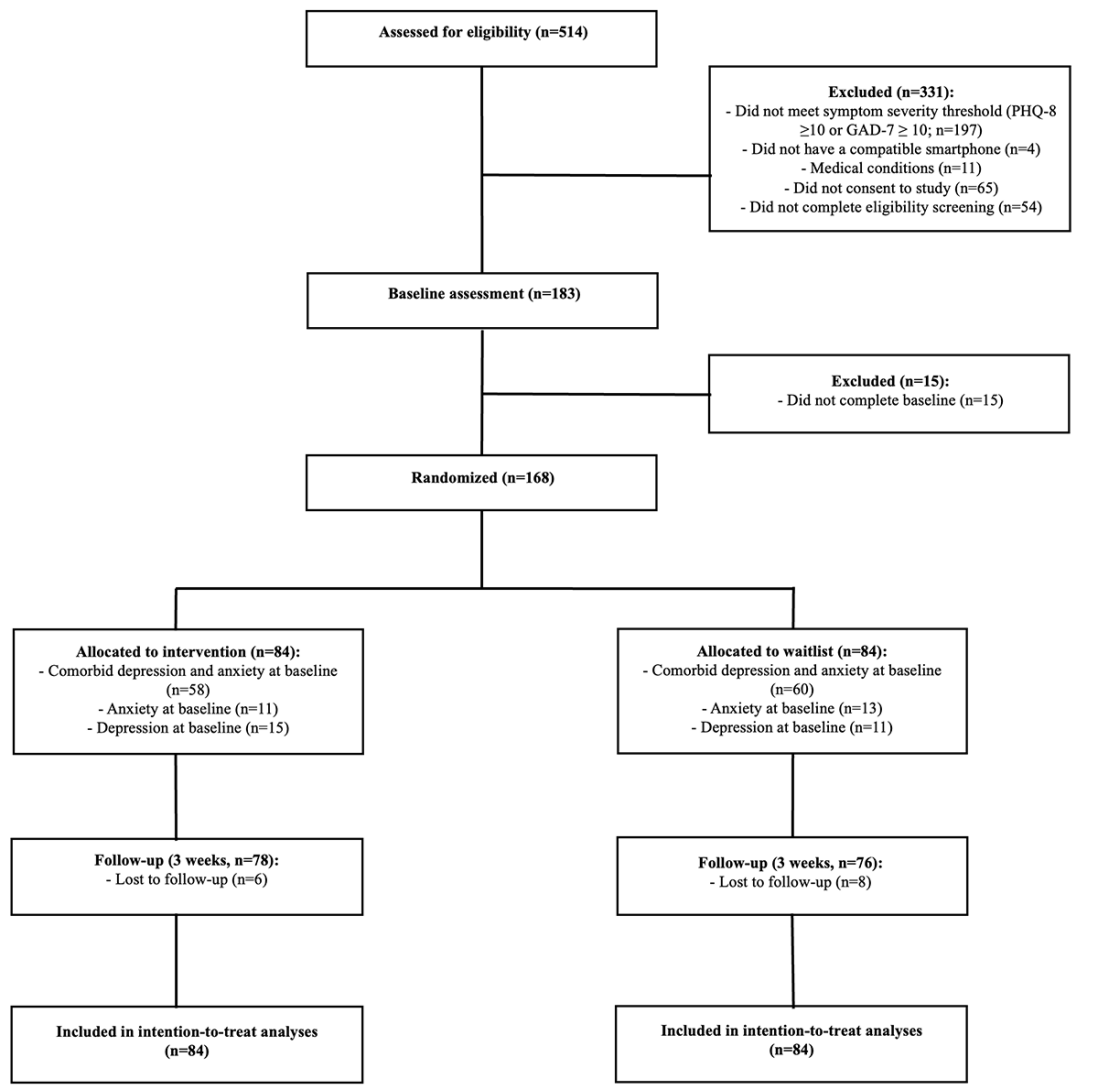
CONSORT diagram and recruitment flowchart. GAD-7: Generalized Anxiety Disorder-7; PHQ-8: Patient Health Questionnaire-8.

A total of 514 potential participants were assessed for eligibility, with 168 participants meeting the inclusion criteria and enrolled in the study (Figure 1). Participants were randomized equally between the intervention group (n=84) and the waitlist control group (n=84). Overall retention was high, with only 14 of 168 (8.3%) participants dropping out before the follow-up assessment (6 from the intervention group and 8 from the waitlist group).

Baseline demographic characteristics were well-balanced between groups (Table 1). The sample consisted predominantly of female participants (intervention: 57/84, 67.9% and control: 60/84, 71.4%), with a mean age of 34.8 (SD 9.6) years in the intervention group and 35.1 (SD 10.7) years in the waitlist control group. The sample was ethnically diverse, with approximately half identifying as White (intervention: 44/84, 52.4% and control: 43/84, 51.2%) and substantial representation of Black (intervention: 18/84, 21.4% and control: 43/84, 23.8%), Asian (intervention: 10/84, 11.9% and control: 8/84, 9.5%), and Hispanic (both groups: 8/84, 9.5%) participants. Most participants were employed (intervention: 60/84, 71.4% and control: 59/84, 70.3%) and had completed at least some college education (intervention: 73/84, 89.3% and control: 74/84, 88.1%). Psychotropic medication use was common at baseline, with 66.7% (56/84) of intervention participants and 63.1% (53/84) of control participants reporting current use. In both groups, the majority reported elevated baseline comorbid anxiety and depression symptoms (intervention: 58/84, 69% and control: 60/84, 71.4%). Psychotropic medication use remained stable from baseline to follow-up in both the intervention and waitlist control groups.

**Table 1:** Baseline demographics by study group of the complete sample.

		Headspace group (n=84)	Waitlist group (n=84)
**Sex, n (%)**			
	Male	27 (32.1)	24 (28.6)
	Female	57 (67.9)	60 (71.4)
Age (years), mean (SD)		34.8 (9.6)	35.1 (10.7)
**Race or ethnicity, n (%)**			
	Asian	10 (11.9)	8 (9.5)
	Black	18 (21.4)	20 (23.8)
	Hispanic or Latino	8 (9.5)	8 (9.5)
	White	44 (52.4)	43 (51.2)
	Other	4 (4.8)	5 (6)
**Employment, n (%)**			
	Employed	60 (71.4)	59 (70.3)
	Unemployed	14 (16.7)	16 (19)
	Retired or disability or other	10 (11.9)	9 (10.7)
**Education, n (%)**			
	High school or less	9 (10.7)	10 (11.9)
	College or associate degree	32 (38.1)	27 (32.1)
	Bachelor’s degree	28 (33.3)	33 (39.3)
	Graduate degree or higher	15 (17.9)	14 (16.7)
**Marital status, n (%)**			
	Married	33 (39.3)	36 (42.9)
	In a relationship	18 (21.4)	17 (20.2)
	Single	23 (27.4)	22 (26.2)
	Divorced	10 (11.9)	9 (10.7)
**Clinical characteristics, n (%)**			
	Both anxiety and depression (baseline PHQ-8^a^≥10 and GAD-7^b^≥10)	58 (69)	60 (71.4)
	Depression only (baseline PHQ-8≥10, GAD-7<10)	15 (17.9)	11 (13.1)
	Anxiety only (baseline GAD-7≥10, PHQ-8<10)	11 (13.1)	13 (15.5)
**Current psychotropic medication**			
	Baseline, n (%)	56 (66.7)	53 (63.1)
	Follow-up^c^, n/N (%)	53/78 (67.9)	50/76 (65.8)

^a^PHQ-8: Patient Health Questionnaire-8.

^b^GAD-7: Generalized Anxiety Disorder-7.

^c^Follow-up percentages calculated from participants who completed the study (intervention: n=78 and control: n=76 after 14 dropouts: 6 intervention and 8 control).

### Anxiety and Depression Symptoms

Both primary outcome measures demonstrated significant interactions between group and time, indicating differential treatment effects (Table 2). Anxiety symptoms (GAD-7) showed a significant group×time interaction (*P*<.001; η^2^_p_=0.350). Follow-up analyses revealed that participants in the intervention group experienced significant reductions in anxiety scores from baseline to postintervention (*P*<.001), baseline to follow-up (*P*<.001), and continued improvement from postintervention to follow-up (*P*=.009). Mean GAD-7 scores in the intervention group decreased from 11.9 (SD 2.8) at baseline to 8.2 (SD 2.6) after the intervention and 7.8 (SD 2.3) at follow-up, representing a 34.5% reduction from baseline (11.9 to 7.8). In contrast, the waitlist control group showed no significant changes across time points, with scores remaining stable around 12.2 at baseline, 11.8 after the intervention, and 11.6 at follow-up. Significant main effects were observed for both time (*P*<.001; η^2^_p_=0.422) and group (*P*<.001; η^2^_p_=0.222).

Depression symptoms (PHQ-8) similarly demonstrated a significant group×time interaction (*P*<.001; η^2^_p_=0.370). The intervention group showed significant reductions from baseline to postintervention (*P*<.001) and baseline to follow-up (*P*<.001), though the change from postintervention to follow-up approached but did not reach significance (*P*=.05). Mean PHQ-8 scores decreased from 12.1 (SD 2.8) at baseline to 8.3 (SD 2.2) after the intervention and 8.0 (SD 2.0) at follow-up in the intervention group, representing a 33.9% reduction from baseline (12.1 to 8.0). The waitlist control group remained stable across all time points (11.8 to 11.4 to 11.3). Significant main effects were found for time (*P*<.001; η^2^_p_=0.472) and group (*P*<.001; η^2^_p_=0.163).

**Table 2:** Primary and secondary outcomes by group and time point for the complete sample.

	Baseline			Postintervention			Follow-up			Model results	
	Headspace group, mean (SD)	Waitlist control, mean (SD)	Headspace group, mean (SD)		Waitlist control, mean (SD)	Headspace group, mean (SD)		Waitlist control, mean (SD)	*P* value		Partial eta squared (η^2^_p_)
Anxiety (GAD-7^a^)	11.9 (2.8)	12.2 (2.6)	8.2 (2.6)		11.8 (2.9)	7.8 (2.3)		11.6 (2.8)	<.001^b^		0.350
Depression (PHQ-8^c^)	12.1 (2.8)	11.8 (2.7)	8.3 (2.2)		11.4 (2.6)	8.0 (2.0)		11.3 (2.9)	<.001^b^		0.370
Sleep quality (PSQI^d^)	11.6 (2.0)	11.6 (1.7)	9.1 (2.2)		11.1 (2.4)	8.1 (2.6)		10.8 (2.3)	<.001^b^		0.172
Perceived stress (PSS-10^e^)	27.2 (3.2)	26.9 (3.1)	25.0 (3.3)		26.9 (2.8)	23.6 (3.4)		26.2 (3.3)	<.001^b^		0.130
Mindfulness (MAAS^f^)	2.9 (0.4)	2.9 (0.3)	3.1 (0.4)		2.9 (0.3)	3.2 (0.5)		3.0 (0.4)	<.001^b^		0.046
Well-being (WEMWBS^g^)	36.4 (8.9)	36.3 (8.1)	37.7 (8.0)		37.1 (7.2)	40.3 (8.1)		37.7 (6.5)	.01^b^		0.030

^a^GAD-7: Generalized Anxiety Disorder-7.

^b^Significant difference between groups (*P*<.05).

^c^PHQ-8: Patient Health Questionnaire-8.

^d^PSQI: Pittsburgh Sleep Quality Index.

^e^PSS-10: Perceived Stress Scale-10.

^f^MAAS: Mindful Attention Awareness Scale.

^g^WEMWBS: Warwick-Edinburgh Mental Well-Being Scale.

### Remission Rates and Treatment Response

Exploratory analyses examining clinically meaningful outcomes revealed significant differences between groups. For remission (defined as scores <5) [20,21,29], GAD-7 remission rates increased significantly in the intervention group over time (*P*=.03), with 11 of 84 (13.1%) participants achieving remission at follow-up compared to 3 of 84 (3.5%) in the waitlist control group (*P*=.12). PHQ-8 remission rates showed improvement in the intervention group (7/84, 8.3%) versus control group (4/84, 4.7%), though this difference was not statistically significant (intervention: *P*=.22 and control: *P*=.41). When combining remission rates across both measures, the intervention group showed significantly higher rates (18/84, 21.4%) compared to controls (7/84, 8.3%; intervention: *P*=.03 and control: *P*=.41).

Treatment response (defined as ≥50% symptom reduction) [20,21,29] also favored the intervention group. GAD-7 response rates increased significantly in the intervention group (*P*=.03), with 9 of 84 (10.7%) participants achieving response at follow-up compared to 1 of 84 (1.1%) in waitlist controls (*P*=.57). PHQ-8 response rates were higher in the intervention group (14/84, 16.6%) versus waitlist controls (1/84, 1.1%; intervention: *P*=.03 and control: *P*=.37). Combined treatment response rates across both measures were significantly higher in the intervention group (23/84, 27.3%) compared to waitlist controls (2/84, 2.3%; intervention: *P*=.03 and waitlist control: *P*=.39).

### Secondary Outcomes

All secondary outcome measures showed significant improvements in the intervention group compared to the waitlist control group (Table 2). Sleep quality (PSQI) demonstrated a significant group×time interaction (*P*<.001; η^2^_p_=0.172). The intervention group showed significant improvements from baseline to postintervention (*P*<.001) and baseline to follow-up (*P*<.001) and continued improvement from postintervention to follow-up (*P*<.001). Mean scores improved from 11.6 (SD 2.0) at baseline to 9.1 (SD 2.2) after the intervention and 8.1 (SD 2.6) at follow-up, representing a 30.2% improvement from baseline. The waitlist control group showed minimal change, though a small improvement from baseline to follow-up was observed (*P*=.04). Main effects were significant for time (*P*<.001; η^2^_p_=0.332) and group (*P*<.001; η^2^_p_=0.149).

Perceived stress (PSS-10) showed a significant group×time interaction (*P*<.001; η^2^_p_=0.130). The intervention group demonstrated significant reductions across all time points: baseline to postintervention (*P*<.001), baseline to follow-up (*P*<.001), and postintervention to follow-up (*P*<.001). Mean scores decreased from 27.2 (SD 3.2) at baseline to 25.0 (SD 3.3) after the intervention and 23.6 (SD 3.4) at follow-up, representing a 13.2% reduction from baseline. No significant changes were observed in the waitlist control group. Significant main effects emerged for time (*P*<.001; η^2^_p_=0.239) and group (*P*=.002; η^2^_p_=0.052).

Mindfulness (MAAS) exhibited a significant group×time interaction (*P*<.001; η^2^_p_=0.046). The intervention group showed significant increases in mindfulness scores from baseline to postintervention (*P*<.001), baseline to follow-up (*P*<.001), and postintervention to follow-up (*P*<.001), improving mean scores from 2.9 (SD 0.4) at baseline to 3.1 (SD 0.4) after the intervention to 3.2 (SD 0.5) at follow-up, representing a 10.3% increase from baseline. The control group remained stable. A significant main effect of time was found (*P*<.001; η^2^_p_=0.176) but not for group (*P*=.05; η^2^_p_=0.023).

Mental well-being (WEMWBS) demonstrated a significant group×time interaction (*P*=.01; η^2^_p_=0.030). The intervention group showed significant improvements from baseline to postintervention (*P*=.02), baseline to follow-up (*P*<.001), and postintervention to follow-up (*P*<.001). Mean scores increased from 36.4 (SD 8.9) at baseline to 37.7 (SD 8.0) after the intervention and 40.3 (SD 8.1) at follow-up, representing a 10.7% increase from baseline. No significant changes occurred in the control group. A significant main effect of time was observed (*P*<.001; η^2^_p_=0.114), but not for group.

### Adherence

Overall retention was high, with 154 of 168 (91.7%) participants completing all 3 study time points. Additionally, adherence to the Headspace Anxiety and Depression Program was high, with participants completing an average of 18.7 (SD 5.5) sessions of the 21 total sessions. The majority of participants (69/84, 82.1%) completed all 21 sessions, and 89.2% (75/84) completed the core elements of the program (ie, 14 sessions where all techniques are introduced). Only 9 (10.7%) participants did not complete the first module (ie, 5 sessions). No significant correlation was found between the number of completed sessions and treatment response across all outcome variables (all *P*s=.14).

### Medication Status

Psychotropic medication use remained stable throughout the study. At baseline, 66.7% (56/84) of intervention participants and 63.1% (53/84) of waitlist control participants reported current medication use. At follow-up, these rates were 67.9% (53/78) and 65.8% (50/76), respectively, with no significant changes in either group. When medication status was included as a covariate in analyses, no significant interactions or main effects were observed for any outcome variable (all *P*s>.15; η^2^_p_<0.010).

### Safety

No serious adverse events were reported during the study. Safety monitoring was conducted through automated flagging in the REDCap platform for clinical deterioration or elevated risk. No technical problems or privacy breaches were reported by participants or detected by the research team. No participants reported difficulty accessing or using the Headspace app interface.

## Discussion

### Principal Findings

This RCT demonstrates that the Headspace Anxiety and Depression Program significantly reduced anxiety and depression symptoms and improved overall well-being in adults with clinically significant anxiety or depression symptoms. Participants in the Headspace group showed significant and sustained improvements across all primary and secondary outcomes, with effect sizes ranging from small to large. The program’s benefits were maintained at a 3-week follow-up, and remission and treatment response rates were significantly higher in the Headspace group versus the waitlist control. While this study did not evaluate outcomes in comparison to an existing intervention, the observed effect sizes for anxiety (η^2^_p_=0.35) and depression (η^2^_p_=0.37) symptom reduction represent large effects and are comparable to established interventions [5,30]. Meta-analytic evidence indicates that teletherapy-delivered CBT achieves similar outcomes to in-person treatment, with effect sizes typically ranging from 0.4 to 0.8 for anxiety and depression symptoms [30].

The 34.5% reduction in anxiety symptoms and 33.9% reduction in depression symptoms observed in our study align with recent meta-analytic findings on DMHIs. Linardon et al [13] reported overall small but significant effects for apps on depression (*g*=0.28) and anxiety (*g*=0.26) symptoms, based on analysis of 176 RCTs examining content-based smartphone interventions incorporating therapeutic elements such as CBT, mindfulness, or behavioral activation. Our intervention demonstrated larger effects, potentially due to the evidence-based, transdiagnostic approach combining CBT and mindfulness techniques focusing on improving emotional regulation. Additionally, the structured, sequential 21-session format ensures comprehensive skill development and consistent engagement. Importantly, 21.4% (18/84) of participants achieved combined remission across both anxiety and depression measures compared to only 8.3% (7/84) in the control group. These remission rates, while modest compared to intensive clinical interventions, represent meaningful recovery for individuals who might otherwise lack access to evidence-based treatment. The demonstrated improvement for participants with both elevated depression and anxiety symptoms also signals the utility of applying a transdiagnostic approach that targets common mechanisms for emotion dysregulation across anxiety and depression. Given the population included in this study (individuals with elevated but not severe anxiety and depression symptoms) and associated improvement, our findings suggest that this program could be a scalable and cost-effective alternative for individuals preferring self-guided treatment or as an adjunct to treatment with a licensed professional (eg, coach, therapist, and psychiatrist).

Beyond primary anxiety and depression outcomes, participants demonstrated significant improvements across all secondary measures, including sleep quality, perceived stress, mindfulness, and overall mental well-being. The improvement in sleep quality is particularly relevant, given the bidirectional relationship between sleep disturbances and mood disorders. Specifically, previous research demonstrates that 50%-80% of individuals with anxiety and depression experience comorbid sleep problems [31]. The increase in mindfulness scores suggests that participants successfully acquired and applied mindfulness skills, which may contribute to long-term resilience and symptom management [32]. These findings are consistent with extensive research on mindfulness-based interventions for anxiety and depression, which typically show moderate effect sizes (anxiety: *d*=0.59 and depression: *d*=0.37) when delivered as stand-alone interventions [33]. The Headspace Anxiety and Depression Program’s integration of mindfulness within a structured CBT framework may have provided participants with both contemplative practice (mindfulness) and practical cognitive tools (CBT) to recognize, understand, and respond to difficult emotions and thoughts. We also note that all mindfulness techniques incorporated into the program were introduced in the first 14 sessions, which 89.2% (75/84) of participants completed. This high engagement may have impacted overall mindfulness outcomes. The improvements in mental well-being are particularly noteworthy, as these measures are typically challenging to influence and represent broader functional improvements beyond symptom reduction.

The high retention (154/168, 91.7%) and adherence rates (69/84, 82.1% completed all sessions) observed in this study are also notable. These rates substantially exceed those typically reported in DMHI research, where adherence rates often range from 50% to 70% [17,28], with Headspace’s similarly designed Sleep Program RCT achieving 72% adherence [34]. Several factors likely contributed to these high engagement rates. The Anxiety and Depression Program’s design emphasized accessibility through brief daily sessions (5-10 minutes), representing a significantly lower time commitment than traditional CBT (typically 50-90 minutes weekly) or even digital CBT programs (often 30-60 minutes per session). Additionally, the sequential structure of the Anxiety and Depression Program, which builds skills progressively across 3 distinct modules, may have enhanced motivation and completion rates. This structured approach contrasts with digital interventions that offer less guided, more self-directed content access. Qualitative data from the follow-up surveys support these reasons for high engagement with participants, indicating that the program being app-based made it very easy to access, the length of the sessions made it doable to fit into their daily schedule, and the program structure making it easy to follow. However, it is important to acknowledge that the financial compensation structure (US $150 total) may have artificially inflated adherence rates, potentially limiting the generalizability of these findings to real-world implementation where such incentives are not provided.

This study’s sample reported high baseline psychotropic medication use (intervention: 56/84, 66.7% and control: 53/84, 63.1%), which remained stable throughout the study. This prevalence exceeds general population estimates of antidepressant use (13.2% in US adults during 2015-2018) [32]. The stability of medication use suggests that the intervention’s benefits were independent of pharmacological changes, suggesting that the intervention can enhance outcomes for individuals already receiving pharmacological care.

### Strengths, Limitations, and Future Research

To our knowledge, this is the first RCT investigating the impact of a self-guided DMHI that uses a transdiagnostic approach with an intentional focus on integrating mindfulness and CBT techniques in a structured, sequential format specifically designed to improve anxiety and depression symptoms. Unlike many digital CBT programs that incorporate mindfulness as a secondary component, the Headspace program was designed with mindfulness as a core therapeutic element. The short daily time commitment, broad commercial availability via an existing app, and the adherence data suggest that the intervention can be practically integrated in a real-world setting. Despite these strengths, the study did have several limitations. First, this study used a waitlist control design, rather than an active control that might provide differential benefits. Second, the relatively short 3-week follow-up period limits conclusions about the long-term durability of treatment effects. Future studies should incorporate longer-term follow-up assessments to evaluate the sustainability of clinical benefits. Third, the moderate baseline symptom severity in our sample may limit the intervention’s applicability to individuals with more severe symptoms. While we excluded participants with severe depression (PHQ-8>20) for safety reasons, future research should examine the program’s effectiveness across the full spectrum of symptom severity. Fourth, while our recruitment approach through social media advertisements may have introduced self-selection bias toward individuals already interested in digital mental health solutions, this limitation is somewhat mitigated by our use of validated screening instruments to confirm clinically significant symptom levels. Fifth, participants were recruited based on validated screening questionnaire scores using self-report symptoms rather than professional clinical diagnoses. While the PHQ-8 and GAD-7 are well-validated screening tools with established clinical cutoffs, they do not replace formal diagnostic evaluation. Future research areas include expanding upon the current RCT to evaluate the effectiveness of the Headspace Anxiety and Depression Program in a real-world setting and examining the use of the program alongside Headspace’s meditation content library and human-delivered care (mental health coaching, therapy, and psychiatry). Future research should also explore how evidence-based DMHIs like the Headspace program might work synergistically with other innovative approaches to well-being, including digital gaming interventions, virtual reality applications, and other media-based therapeutic tools [33]. The integration of multiple digital modalities may offer personalized pathways to mental health improvement.

### Conclusions

The Headspace Anxiety and Depression Program represents an efficacious, accessible, and scalable approach to mental health treatment for individuals with clinically significant anxiety and depression symptoms. The transdiagnostic intervention consisting of 21 daily brief 5- to 10-minute sessions demonstrated substantial improvements across multiple domains of mental health and well-being, with benefits maintained at follow-up. These findings support the potential for evidence-based DMHIs to address critical gaps in mental health care delivery, particularly for populations who face barriers accessing traditional treatment modalities or have a preference for self-guided care.

## Appendix

Multimedia Appendix 1 CONSORT-eHEALTH checklist (V 1.6.1).

Multimedia Appendix 2 Differences between the Headspace Anxiety and Depression Program, other digital cognitive behavioral therapy programs, and traditional cognitive behavioral therapy.

Multimedia Appendix 3 Headspace Anxiety and Depression Program session outline and descriptions.

Multimedia Appendix 4 Headspace Anxiety and Depression Program within the Headspace app.

## Abbreviations

CBT: cognitive behavioral therapy
CONSORT: Consolidated Standards of Reporting Trials
DMHI: digital mental health intervention
GAD-7: Generalized Anxiety Disorder-7
MAAS: Mindful Attention Awareness Scale
PHQ-8: Patient Health Questionnaire-8
PSQI: Pittsburgh Sleep Quality Index
PSS-10: Perceived Stress Scale-10
RCT: randomized controlled trial
REDCap: Research Electronic Data Capture
UP: Unified Protocol
WEMWBS: Warwick-Edinburgh Mental Well-Being Scale

## Data Availability

The deidentified datasets generated and analyzed during the study are available from the corresponding author on reasonable request.
