# Supplementary material for: Efficacy of a Self-Guided Transdiagnostic Intervention for Adults With Anxiety and Depression: Randomized Controlled Trial
Source: JMIR Mhealth Uhealth. 2025 Oct 23;13:e79759. doi: 10.2196/79759 (PMC12592896; doi:10.2196/79759)
Supplement: Multimedia Appendix 2 [file mhealth_v13i1e79759_app2.docx]

|  | Headspace Anxiety and Depression Program | Digital CBT programs | Traditional CBT (in person and teletherapy) |
| --- | --- | --- | --- |
| Overview | Guided program (current study) dedicated to improving anxiety and depression symptoms | Other digital programs with CBT techniques | In person or digital CBT/therapy led by a therapist/counselor |
| What is it? | Self-guided program starting with mindfulness tools to develop awareness of thoughts and behaviors that shape emotions; CBT tools to practice changing emotional responses | Programs incorporating digital therapy and/or coaching combined with self-guided tools and content | Explore underlying causes, learn coping strategies, and develop healthier thought patterns and behaviors |
| How often? | 21 consecutive, daily 5-10 minute sessions | 1-6 months | 12-20+ weekly sessions (can be up to several years) |
| Who leads? | Self-guided, content led by a Headspace mindfulness teacher, psychiatrist, and Headspace members | Digital therapist/coach | In person/digital therapist/counselor |
| Where is it located? | Headspace app | Other digital apps | In person/digital |
| What is the cost? | Headspace membership  *$12.99/month or $69.99/year* | Membership cost (varies)  *$99-$600/year* | Per session cost  *Up to $250/session depending on insurance* |
| Who is the target? | Anyone, but designed to deliver outcomes for subclinical/clinical anxiety and/or depression symptoms | Anyone with anxiety or depression symptoms | Clinical levels of anxiety or depression |
| Abbreviations: CBT (cognitive behavioral therapy) | | | |
