# Supplementary material for: Efficacy of a Self-Guided Transdiagnostic Intervention for Adults With Anxiety and Depression: Randomized Controlled Trial
Source: JMIR Mhealth Uhealth. 2025 Oct 23;13:e79759. doi: 10.2196/79759 (PMC12592896; doi:10.2196/79759)
Supplement: Multimedia Appendix 3 [file mhealth_v13i1e79759_app3.docx]

| **Session** | **Session objective** |
| --- | --- |
| Part 1: Explore | Get to know how you usually react to your emotions. From there, you can see where you might want to make changes |
| Session 1 | Get started with the Anxiety and Depression Program, understand your “why”, and give yourself a reminder to come back to it every day. |
| Session 2 | Recognize how strong emotions show up for you. |
| Session 3 | Learn why feeling better starts with awareness. |
| Session 4 | Explore how you react to strong emotions. |
| Session 5 | Recognize how cloudy thoughts affect your mood. |
| Part 2: Experiment | Try CBT tools and techniques to cope with your emotions, and find out which ones work best for you. |
| Session 6 | Explore what behaviors help your response to emotions. |
| Session 7 | Find ways to boost your mood and bring more joy into everyday life. |
| Session 8 | Learn how to move through avoidance and procrastination. |
| Session 9 | Learn how to ground yourself through tough moments. |
| Session 10 | Uncover helpful thoughts and take a look at things from a new perspective. |
| Session 11 | Recognize thinking patterns and get to know why your mind sometimes gets “stuck.” |
| Session 12 | Notice how emotions physically feel in your body. |
| Session 13 | Learn how to prevent worries from clouding your day. |
| Session 14 | Setting a personal challenge to feel happier. |
| Part 3: Maintain | Learn how to keep up the changes you’ve made, so you can handle anything that comes your way. |
| Session 15 | Now that you’ve made the changes, learn to make them stick. |
| Session 16 | Learn how to solve problems that might come up along your journey. |
| Session 17 | Remembering your “why,” keep yourself motivated as you work toward your goals. |
| Session 18 | Building your support system to get help from the people around you. |
| Session 19 | Find out what to do when things don’t go as planned. |
| Session 20 | Make a plan for what to do when you encounter setbacks. |
| Session 21 | Now that you’ve completed the program, what’s next to continue your journey? |
