# Supplementary figures and images for: Efficacy of a Self-Guided Transdiagnostic Intervention for Adults With Anxiety and Depression: Randomized Controlled Trial
Source: JMIR Mhealth Uhealth. 2025 Oct 23;13:e79759. doi: 10.2196/79759 (PMC12592896; doi:10.2196/79759)

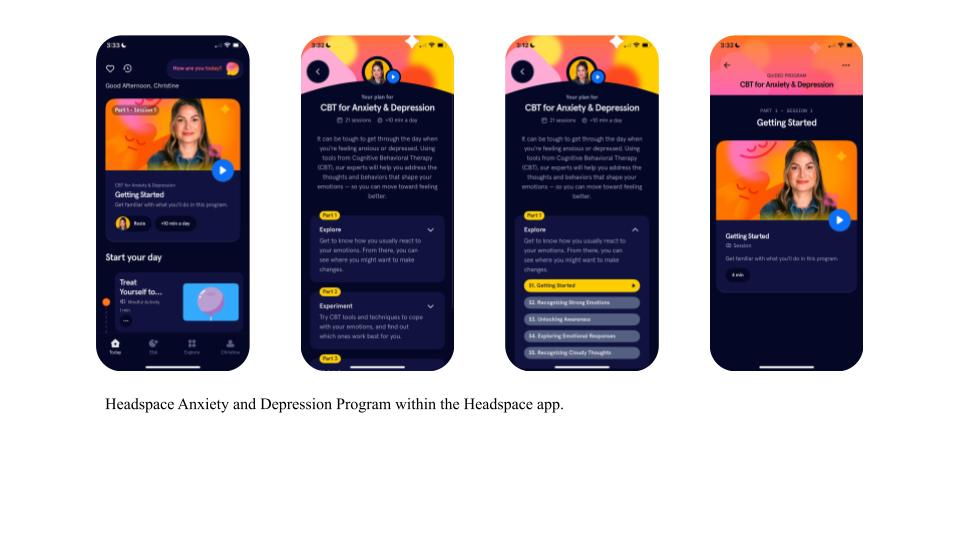

Supplement: Multimedia Appendix 4 [file mhealth_v13i1e79759_app4.png]
